# Explainable Transformer-Based Modelling for Pathogen-Oriented Food Safety Inspection Grade Prediction Using New York State Open Data

**DOI:** 10.3390/foods15020223

**Published:** 2026-01-08

**Authors:** Omer Faruk Sari, Mohamed Bader-El-Den, Volkan Ince

**Affiliations:** 1 School of Computing, University of Portsmouth, Lion Terrace, Portsmouth PO1 3HE, UK; omer.sari@port.ac.uk (O.F.S.); volkan.ince@port.ac.uk (V.I.); 2 College of Computer and System Engineering, Abdullah Al Salem University (AASU), Kuwait City Firdous Street, Block 3 Khaldiya, Kuwait City 72303, Kuwait

**Keywords:** food safety inspections, pathogen detection, real-time risk assessment, explainable artificial intelligence

## Abstract

Foodborne pathogens remain a major public health concern, and the early identification of unsafe conditions is essential for preventive control. Routine inspections generate rich textual and structured data that can support real-time assessment of pathogen-related risk. The objective of this study is to develop an explainable transformer-based framework for predicting food safety inspection grades using multimodal inspection data. We combine structured metadata with unstructured deficiency narratives and evaluate classical machine learning models, deep learning architectures, and transformer models. RoBERTa achieved the highest performance (F1 = 0.96), followed by BiLSTM (F1 = 0.95) and LightGBM (F1 = 0.92). SHapley Additive exPlanations (SHAP) analysis revealed linguistically meaningful indicators of pathogen-related hazards such as temperature abuse, pests, and unsanitary practices. The findings demonstrate that transformer-based models, combined with explainable AI (XAI), can support pathogen-oriented monitoring and real-time risk assessment. This study highlights the potential of multimodal AI approaches to enhance inspection efficiency and strengthen public health surveillance.

## 1. Introduction

Foodborne illnesses represent a significant global public health challenge, affecting approximately 600 million people annually and causing 420,000 deaths, with economic losses exceeding 100 billion dollars [1]. Accurate estimation of their true burden is hampered by the inconsistent quality of surveillance across countries, unequal access to healthcare, inadequate reporting, diagnostic hurdles, and the different clinical presentations of these diseases [2,3]. Although these figures are subject to some uncertainty, they emphasise that foodborne diseases remain one of the leading causes of death, particularly in low and middle income regions. To address this threat, many governments have enacted comprehensive food safety regulations and surveillance programmes, with routine inspections of food establishments being a cornerstone of these efforts. During these visits, inspectors verify compliance with hygiene standards and food handling protocols and work with operators to identify risks and develop practical solutions [4].

Within this comprehensive regulatory framework, United State (U.S) food retailers play a prominent role in protecting consumers. With over six million employees and nearly 800 billion dollars in annual sales, retailers are responsible for the safety of all the products they sell. Their responsibilities range from auditing supplier safety practices to implementing rigorous in-store food safety programs and even extend to educating customers about proper handling and storage. Consumer confidence is high: according to surveys, 93 percent of American shoppers believe that retailers adhere to safety standards and remove products immediately in the event of recalls or outbreaks [5,6].

Despite these robust monitoring mechanisms and the high level of public confidence, most retailers continue to rely on traditional laboratory tests such as gas chromatography, high-performance liquid chromatography and polymerase chain reaction to verify food safety. While these methods offer high analytical accuracy, they are destructive, laborious and costly, and often take hours or days to provide results. These limitations make them poorly suited for real-time or in-line quality control in modern, high-throughput food processing environments where rapid, non-invasive assessment is essential to protect public health and maintain operational efficiency [7,8].

Food safety inspection grades are strongly associated with conditions conducive to pathogen growth, including improper temperature control, cross-contamination, inadequate sanitation, and pest activity. Although inspectors do not routinely perform microbiological testing during routine visits, these environmental and procedural violations are established predictors of contamination by *E. coli*, *Salmonella*, *Listeria monocytogenes*, and other foodborne pathogens [9]. Prior work has shown that temperature abuse and sanitation failures correlate with elevated microbial loads and increased outbreak risk. Traditional pathogen detection methods such as culture techniques, biochemical assays, and Polymerase Chain Reaction (PCR) offer high analytical precision but are slow, destructive, labor-intensive, and unsuitable for real-time decision-making. As a result, health authorities rely heavily on visual inspection data as a proxy for microbial safety. Machine learning (ML) provides a scalable complement to laboratory diagnostics, enabling real-time pathogen-risk inference from multimodal inspection data. By interpreting inspection narratives and structured features, AI systems can identify linguistic and operational indicators of microbial hazards and support early intervention [10].

The proliferation of large scale data across sectors such as industry, healthcare, government, and research fields ranging from the natural and life sciences to the social sciences, humanities, and engineering offers unprecedented opportunities to generate insights, inform decision-making, and optimize products and services. In the US, Data.gov [11], established by the OPEN Government Data Act, provides a central repository of standardised, machine-readable datasets to improve transparency and accountability. Complementing this resource, platforms such as the Rapid Alert System for Food and Feed (RASFF) [12], the United States Department of Agriculture (USDA) [13], the Food Standards Agency (FSA) [14] and the European Food Safety Authority [15] curate and disseminate daily food safety data that underpins evidence-based policy, facilitates scientific collaboration, and enables advanced analytical approaches to food safety management.

Food safety inspections have traditionally relied on visual assessment of production facilities and handling procedures to ensure compliance with regulatory standards. However, the explosion of inspection data requires advanced analytical techniques capable of uncovering predictive patterns at scale. Recent advances in artificial intelligence, from machine learning [16,17] to deep learning (DL) architectures [18,19], have shown promise in modeling complex, heterogeneous data; moreover, the advent of XAI methods [1] enables practitioners to understand and trust model outputs. These methods, which improve both the speed and precision of data analysis, enable prompt risk assessments and support evidence-based strategies in food safety monitoring.

Despite the central role that food inspection plays in protecting public health, there remains a notable lack of research that applies advanced machine learning, deep learning and transformer based modelling to this domain particularly approaches that integrate interpretability to support transparency, accountability and practitioner trust. Previous studies [20,21,22,23,24] have explored different perspective of food safety and inspection, they have largely overlooked the important role of interpretability and transparency enabled by XAI.

To address the limitations of conventional risk assessment approaches, we introduce an interpretable deep learning framework trained on U.S. food inspection records. Our approach leverages a multi-input neural network that integrates structured data including historical scores, violation counts, operational characteristics, and temporal factors with transformer-based embeddings of inspection narratives. By incorporating SHAP based explainability directly into the model, we enable both global insights into key predictors and granular, audit-specific explanations that reveal the factors driving individual grade predictions.

The remainder of this paper is structured as follows. Section 2 reviews related work, summarizing key approaches along with their strengths and limitations. Section 3 describes the dataset, preprocessing, model architecture, and analytical methods. Section 4 presents the experimental results, which are interpreted and discussed in Section 5. Finally, Section 6 concludes the paper and outlines directions for future research.

## 2. Related Works

Food safety inspection prediction has gained growing attention in recent years, particularly with the availability of open government datasets and the advancement of machine learning methods. Early studies largely relied on traditional statistical models and basic classification algorithms to predict inspection outcomes. A recent study [1] proposed a comprehensive AI-driven framework that integrates traditional machine learning techniques, deep learning models such as Long Short-Term Memory (LSTM), and advanced transformer architectures like Bidirectional Encoder Representations from Transformers (BERT) and RoBERTa to analyze the European Union (EU)’s RASFF dataset. By incorporating data enrichment strategies and XAI methods particularly SHapley Additive exPlanations (SHAP) the study achieved classification accuracies of up to 97–98%, highlighting both the superior predictive capabilities of transformer-based models and the critical role of XAI in uncovering domain-specific risk factors.

Researchers [25] explored the use of XAI for predicting food fraud risks using deep learning models trained on data from the RASFF and European Medicines Agency databases. By applying interpretability tools such as SHAP, Local Interpretable Model-agnostic Explanations (LIME), and the What-If Tool, the study demonstrated both the potential and current limitations of XAI in food safety applications. While their focus was on fraud detection, their findings highlight the importance of transparency in AI systems an issue our work addresses in the context of food safety inspection grading.

Researchers [26] addressed the challenge of improving food import inspection efficiency in South Korea by developing a soft voting ensemble model to predict non-conformance in imported seafood inspections. Their approach tackled severe class imbalance using cost-sensitive learning and combined classifiers such as Decision Trees, Random Forests, Logistic Regression, and Naive Bayes. The ensemble achieved strong predictive performance (AUC = 87.49%) and incorporated SHAP analysis to interpret key factors influencing predictions such as seasonality, exporter country, and importer characteristics. This work highlights the value of interpretable ensemble models in regulatory food inspection contexts and demonstrates the broader applicability of XAI to public health and safety challenges.

A recent study by [27] proposed a multimodal deep learning system for food safety monitoring, integrating computer vision, Natural Language Processing (NLP), and sensor data. The system employed Swin Transformers for defect detection and temporal convolutional networks for predicting storage conditions. With blockchain and federated learning for secure data sharing, the model achieved over 98% accuracy in detecting contamination and monitoring supply chain anomalies, demonstrating the potential of intelligent, end-to-end AI systems in enhancing food safety regulation.

Authors [28] analysed over 14,000 food inspection records from China, focusing on chemical hazards such as pesticides, heavy metals, and antibiotics. Using data mining techniques, specifically the Apriori algorithm, they identified frequent associations between hazardous substances and specific food categories (e.g., antibiotics in fish, pesticides in vegetables) to enable early warning alerts. Their work demonstrated the potential of association rule mining to extract risk patterns from inspection data, but did not explore advanced machine learning, deep learning architectures, or XAI methods.

Researchers [29] present a machine vision-based system for food quality inspection and grading, focusing on fruits such as apples and mangoes. Their approach combines image preprocessing (Gaussian filtering for noise removal and histogram equalization for contrast enhancement), K-means clustering for image segmentation, and machine learning classifiers (KNN, SVM, and C4.5) for determining fruit quality (good vs. rotten). Their experiments on a small dataset of 250 images demonstrated that SVM achieved the highest accuracy (98%), outperforming KNN and C4.5 across accuracy, sensitivity, and specificity metrics. The study highlights the potential of image processing and traditional ML algorithms for automating visual inspection tasks but does not address structured tabular data or interpretability challenges, nor does it explore modern deep learning architectures like transformers.

A recent study [30] developed a ML framework to predict food safety compliance of food outlets in England and Wales, motivated by declining inspection rates and resource constraints in UK local authorities. The authors hypothesized that food outlet compliance is linked to neighborhood characteristics, and they trained classification models using publicly available data on sociodemographics, urbanness, deprivation indicators, and business type. They tested linear SVM, radial SVM, and random forest classifiers on a dataset of over 300,000 food outlets, addressing class imbalance (only 7% non-compliance) using undersampling and SMOTE. The best-performing model a random forest trained with SMOTE data at a 1:1 ratio achieved 84% sensitivity in identifying non-compliant outlets. The study highlights that local socioeconomic context and demographic factors are predictive of food safety compliance, and it demonstrates the potential of neighborhood-level data for prioritizing inspections. However, the work did not include deep learning methods or interpretability frameworks, and predictive features were limited to structured aggregate data without incorporating text or operational history.

Taken together, these studies illustrate the evolution of food safety inspection prediction from traditional statistical analysis and data mining toward sophisticated multimodal and XAI systems. While previous research has demonstrated the utility of machine learning and data mining methods to identify patterns in food inspection results, much of this work has either focused on narrow domains (e.g., chemical hazards, image-based classification) or used classical algorithms with limited focus on model interpretability. Recent advances, particularly those utilising transformer architectures, multimodal integration and XAI methods such as SHAP, highlight the growing potential of AI to both improve predictive accuracy and support transparency and accountability in regulatory decision-making. However, there are still gaps in systematically comparing modern deep learning models (e.g., transformers) with traditional machine learning approaches on large, structured inspection datasets, while embedding interpretability into prediction frameworks. Our work builds on these gaps and proposes an interpretable deep learning model that integrates structured tabular features with transformer-based textual representations specifically tailored to the challenges of assessing large food safety inspection datasets, providing the inspector with ready-to-use explanations that promote confidence and actionable insights.

## 3. Materials and Methods

**Figure 1:**
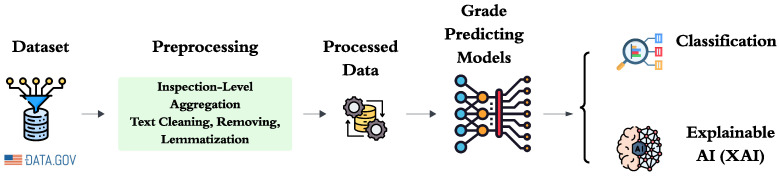
End-to-end pipeline for explainable food-safety inspection grade prediction. Raw inspection records are first aggregated at the inspection level, merging all deficiency entries for each visit. Next, free-text descriptions undergo cleaning (lowercasing, punctuation removal, stop-word filtering) and lemmatization, while structured metadata (e.g., past grades, violation counts, establishment attributes, temporal features) are standardized and encoded. The processed data then feeds into a suite of classification models ranging from traditional machine-learning algorithms to multi-input deep and transformer-based networks to predict inspection grades. Finally, explainable AI techniques (SHAP attributions and attention visualizations) are applied to illuminate both global and case-specific factors driving each model output.

In this section, we present our end-to-end framework for predicting food safety inspection scores. It begins by ingesting raw inspection datasets and perform rigorous data cleaning, aggregation, and feature extraction, capturing both structured metadata and textual embeddings of deficiency descriptions. Then train and evaluate a range of classifiers, ranging from traditional machine learning algorithms to deep learning networks and transformer-based models, each tuned through cross-validation. Finally, integrate XAI methods such as SHAP attributions and attention visualisations to decipher the decision-making process and highlight the most influential features that affect each prediction. Figure 1 presents a schematic of our end-to-end framework, detailing each stage from data ingestion and inspection-level aggregation through feature extraction, multi-input model inference, and the application of explainability techniques.

### 3.1. Preprocessing

Preparing the Food Safety Inspections dataset for analysis involved several sequential steps to ensure consistency, integrity, and suitability for both modelling and mapping. First, the raw csv file was ingested, and all column names were converted to lowercase with underscores replacing spaces to facilitate programmatic handling. Inspection dates, originally stored as strings in “mm/dd/yy” format, were parsed into date time objects, from which month, year, and season (spring, summer, autumn, winter) indicators were derived to support temporal analyses and seasonality modelling.

Next, the geospatial information encoded in Well-Known Text (WKT) as “POINT (longitude latitude)” was extracted into numeric longitude and latitude columns. These were loaded into a GeoPandas GeoDataFrame (EPSG:4326), and records with missing or malformed coordinates were excluded to preserve mapping accuracy. Categorical fields such as county, city, and establishment_types were examined for infrequent levels (fewer than 0.5% of records) and those rare categories were grouped into an “other” bucket; all categories were then one-hot encoded for use in machine learning pipelines.

To capture the presence of violations, a binary deficiency flag feature was created, marking inspections with any non-null deficiency description. The free-text deficiency descriptions themselves were lowercased, stripped of punctuation and special characters, and tokenized in preparation for downstream NLP tasks; entries without text were retained as null tokens to maintain alignment with structured records. Numerical features (month, deficiency_flag) were left in their native form, while missing values in other numeric or categorical columns were imputed using median (for numeric) or mode (for categorical) strategies when column completeness exceeded 90%; columns with greater than 10% missingness were dropped for modelling purposes.

Finally, the fully processed dataset was split via stratified sampling into training (80%) and testing (20%) subsets, preserving the proportional representation of inspection grades (A, B, C). This stratification ensures that model evaluation reflects the real-world distribution of inspection outcomes and supports reliable comparisons across different classification and text-enhanced modeling approaches. The cleansed dataset represents a high quality, harmonised inventory of food safety inspection events containing valid, complete records with standardised feature coding and careful handling of missing items. This rigorous cleaning process ensured that subsequent analyses and model training would reflect true patterns in the inspection results, free from artefacts caused by data quality issues. The resulting dataset provided a solid foundation for the development of an interpretable deep learning framework that could incorporate both structured attributes (historical scores, number of violations, operational characteristics) and unstructured textual features (deficiency descriptions), while providing inspectors with ready-to-use explanations aligned with public health requirements.

### 3.2. Data Quality Assessment and Processing

Open government datasets often contain noise, missing information, and inconsistent annotations. To ensure analytical reliability, a comprehensive data quality assessment was performed prior to modeling. Each attribute was evaluated for completeness. Columns with more than 10% missingness were removed, while numerical variables with <10% missing values were imputed using median values and categorical variables using mode frequency. Before quality processing, the raw dataset exhibited an integrity rate of 88.9%, primarily due to missing or incomplete fields, and a consistency rate of 91.7% caused by duplicated or irregular entries. After applying the quality-processing pipeline, the resulting dataset achieved an overall integrity rate of 97.4% and an improved consistency rate of 98.1%. Inconsistent category labels (e.g., “restuarant,” “restraunt”) were merged through frequency-based normalization. Extremely rare categories (<0.5% occurrence) were grouped into an “Other” class to reduce sparsity. Location fields encoded in Well-Known Text were validated and converted to coordinate pairs, with malformed entries discarded. Inspection grades and deficiency codes were harmonized to ensure uniformity across jurisdictions and time periods. This rigorous quality-processing pipeline ensures high-fidelity inputs for model training and prevents artifacts arising from noisy or incomplete records.

### 3.3. Classification Models

In the classification stage, a wide spectrum of learning paradigms was explored, encompassing traditional machine learning algorithms, deep learning architectures, and state-of-the-art transformer-based models. The aim was to systematically compare modeling strategies capable of handling both structured metadata and unstructured textual descriptions typical of food inspection records.

**Machine Learning Models:** Several classical machine learning models were implemented as baselines, including Logistic Regression, Support Vector Machines (SVM), Decision Trees, Random Forests, Naive Bayes, AdaBoost, XGBoost, LightGBM, and a shallow Artificial Neural Network (ANN). These models operate on structured tabular features and vary in their underlying assumptions and learning mechanisms. Tree-based ensembles such as Random Forests and boosting frameworks like XGBoost and LightGBM are particularly suited for handling high-dimensional, heterogeneous features and are capable of capturing complex non-linear interactions. Linear models like Logistic Regression and SVM provide interpretable baselines, while Naive Bayes offers a probabilistic approach based on conditional independence assumptions.

**Deep Learning Architectures:** To extend modeling capacity beyond structured inputs, Deep learning models capable of processing textual data were incorporated into the framework. Convolutional Neural Networks (CNNs) [31] were used to detect localized patterns and phrase-level semantics within the inspection narratives. CNNs apply discrete convolution operations of the following form:

$$\left(f*g\right)\left(t\right)=\sum_{i=0}^{n}f\left(i\right)\;g\left(t-i\right),$$

where *f* is the input sequence, *g* the convolutional kernel, and *t* the position in the sequence. Activation functions and pooling layers aggregate these local features into global representations suitable for classification.

Recurrent models were also employed to capture sequential dependencies. Long Short-Term Memory (LSTM) [32] units maintain memory cells governed by input, forget, and output gates:

(1) $$\begin{array}{cc} f_{t} & =\sigma (W_{f}\left[h_{t-1},x_{t}\right]+b_{f}), \end{array}$$

(2) $$\begin{array}{cc} i_{t} & =\sigma \left(W_{i}\left[h_{t-1},x_{t}\right]+b_{i}\right),\;{\tilde{C}}_{t}=\tanh\left(W_{C}\left[h_{t-1},x_{t}\right]+b_{C}\right), \end{array}$$

(3) $$\begin{array}{cc} o_{t} & =\sigma (W_{o}\left[h_{t-1},x_{t}\right]+b_{o}), \end{array}$$

(4) $$\begin{array}{cc} C_{t} & =f_{t}⊙C_{t-1}+i_{t}⊙{\tilde{C}}_{t}, \end{array}$$

(5) $$\begin{array}{cc} h_{t} & =o_{t}⊙\tanh\left(C_{t}\right), \end{array}$$

where σ and tanh denote sigmoid and hyperbolic tangent activations, respectively. Bidirectional LSTMs (BiLSTMs) extend this design by processing sequences in both forward and backward directions to capture full contextual information:

$$h_{t}=\left[{\vec{h}}_{t};\;{\overset{\leftarrow }{h}}_{t}\right].$$

Gated Recurrent Units (GRUs) [33] offer a simplified variant by combining gates and streamlining cell updates:

(6) $$\begin{array}{cc} z_{t} & =\sigma (W_{z}\left[h_{t-1},x_{t}\right]+b_{z}), \end{array}$$

(7) $$\begin{array}{cc} r_{t} & =\sigma (W_{r}\left[h_{t-1},x_{t}\right]+b_{r}), \end{array}$$

(8) $$\begin{array}{cc} {\tilde{h}}_{t} & =\tanh(W_{h}\left[r_{t}⊙h_{t-1},x_{t}\right]+b_{h}), \end{array}$$

(9) $$\begin{array}{cc} h_{t} & =\left(1-z_{t}\right)⊙h_{t-1}+z_{t}⊙{\tilde{h}}_{t}. \end{array}$$

**Transformer-Based Models:** The framework was further extended to include transformer-based architectures, which model full-sequence dependencies without recurrence by using self-attention mechanisms. The core operation in transformers is defined as

(10) $$Attention\left(Q,K,V\right)=\mathrm{softmax}\left(\frac{QK^{⊤}}{\sqrt{d_{k}}}\right)V$$

where *Q*, *K*, and *V* are query, key, and value matrices, and $d_{k}$ is the dimensionality of the key vectors. This formulation allows for each token in the sequence to attend to all others, capturing both local and global context simultaneously. Models based on BERT employ bidirectional self-attention to encode rich contextual representations. In contrast, GPT uses unidirectional (causal) attention, processing inputs left-to-right. RoBERTa enhances BERT with dynamic masking and optimized pretraining; XLNet employs permutation-based learning; and BioBERT incorporates domain-specific corpora.

All deep learning and transformer models were trained using early stopping to prevent overfitting, with a maximum of 100 epochs and a patience threshold of five validation steps. Transformer models were fine-tuned on the corpus of deficiency narratives, leveraging their pretrained language representations to capture semantic nuances critical for inspection-grade prediction.

**Implementation Framework:** All models were implemented in Python using widely adopted machine learning libraries. Classical algorithms were developed with scikit-learn [34]; deep neural networks with TensorFlow and the Keras API [35,36]; and transformer architectures were fine-tuned and evaluated using Hugging Face Transformers [37]. This unified software stack ensured reproducibility, consistent data preprocessing, and systematic evaluation across all modeling approaches. Also, All experiments were performed in Google Colab using Python 3.10, CUDA 12.2, and cuDNN 8.9. Deep learning models were implemented with TensorFlow 2.15 and PyTorch 2.1, while transformer architectures were fine-tuned using HuggingFace Transformers 4.37. Classical machine learning models were developed using scikit-learn 1.3. The cloud environment provided access to either an NVIDIA Tesla T4 (16 GB VRAM) or NVIDIA A100 (40 GB VRAM) GPU, along with an Intel Xeon 2.20 GHz CPU and 12–25 GB RAM. Transformer models differ substantially in their computational cost. BERT-base contains 110 M parameters, while RoBERTa-base contains 125 M parameters due to additional training optimizations. XLNet-base includes 117 M parameters but requires higher memory bandwidth because of its permutation-based training objective. In comparison, classical ML models contain fewer than 1 M parameters, and deep neural networks (e.g., BiLSTM) typically contain 5–10 M parameters. Training time followed the same pattern: classical ML < deep learning < transformer models. RoBERTa showed the highest training cost but also delivered the strongest performance, indicating a clear accuracy cost trade-off.

#### Model Explainability

XAI is essential for opening the “black box” of modern predictive models, especially in sensitive areas such as food-safety risk assessment. Although deep neural networks and Transformer-based architectures often achieve state-of-the-art accuracy, their internal mechanics remain opaque, which can undermine stakeholder confidence and complicate regulatory compliance. XAI methods counteract this by revealing which inputs drive each prediction, thereby supporting transparency, accountability, and broader adoption in high-stakes contexts.

In the realm of food safety, this interpretability is invaluable: inspectors, regulators, and business operators need to understand whether a high-risk flag stems from, for example, a particular contamination indicator, an establishment’s historical record, or regional environmental factors. The key benefits of introducing XAI into our risk-prediction framework include:

- **Regulatory Accountability**: Every model decision can be traced back to its most influential features, facilitating audits and justifications required by public-health authorities.
- **User Confidence and Adoption**: Providing clear, human-readable explanations makes automated tools more palatable to end users, increasing buy-in from industry and policymakers.
- **Bias and Error Detection**: By highlighting unexpected feature contributions, XAI helps uncover model weaknesses or dataset imbalances, leading to fairer and more dependable predictions.

Among XAI methods, SHAP (SHapley Additive exPlanations) stands out for its rigorous foundation in cooperative game theory, offering a fair allocation of importance to each feature. The SHAP value for feature *i* is computed as

(11) $$\phi_{i}=\sum_{S\subseteq N\setminus \{i\}}\frac{|S|!(|N|-|S|-1)!}{|N|!}\left[f(S\cup \{i\})-f(S)\right]$$

Here:

- $\phi_{i}$: The SHAP value for feature *i*, representing its contribution to the prediction.
- *N*: The set of all input features.
- *S*: A subset of features excluding *i*.
- f(S): The model’s prediction for the subset *S*.

This formulation guarantees that the summed contributions of all features equal the total difference between the model’s prediction for the complete input and its expectation over the feature distribution, ensuring both local fidelity and global consistency.

**Figure 2:**
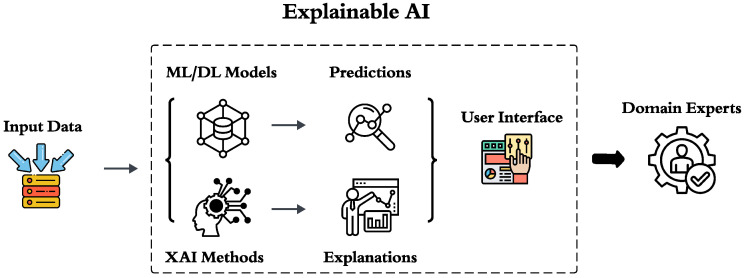
Schematic overview of the Explainable AI framework for inspection-grade prediction. Raw inspection data are ingested and processed by machine-learning/deep-learning (ML/DL) models to generate initial grade predictions. These predictions are then interpreted through XAI methods to produce feature-level explanations. A user interface conveys both predictions and explanations to domain experts, ensuring transparency and facilitating informed decision-making in food safety risk assessment.

Figure 2 demonstrates the steps of XAI.

## 4. Results

### 4.1. Overview of the Food Safety Inspections Dataset

The Food Safety Inspections dataset used in this study contains 143,601 individual inspection records collected by the New York State Department of Health between March 2023 and March 2025. Each record corresponds to a single inspection event, resulting in one of three possible grades A, B, or C based on observed compliance with sanitary regulations.

The dataset spans a wide geographical area across New York State, encompassing both urban and rural regions. The highest concentration of inspections occurs in the metropolitan areas of New York City, including Kings, Queens, Bronx, and New York counties, which together account for more than 70% of all inspections. Additional dense clusters appear in Erie and Monroe counties, reflecting the concentration of food service establishments in these population centers.

Each dataset entry contains a rich combination of structured and unstructured attributes:

- **County, City, Zip Code**: Geographic identifiers of each establishment.
- **Georeference**: Point geometry encoded in Well-Known Text (WKT) format (EPSG:4326), later transformed into numeric longitude–latitude coordinates for mapping and geospatial analysis.
- **Inspection Date**: The date of each inspection, converted to a standardized datetime object for temporal analysis and seasonality modelling.
- **Inspection Grade**: The categorical outcome (A, B, or C) reflecting overall compliance.
- **Establishment Type**: A coded descriptor of the facility category (e.g., “AC” for restaurant/deli, “A” for market).
- **Deficiency Number and Description**: Violation identifiers and corresponding narrative text detailing the nature of non-compliance.

Overall, this dataset captures a heterogeneous sample of food establishments varying in business type, geographic location, and compliance level. The coexistence of structured features (e.g., location, establishment category, date) and unstructured narratives (e.g., deficiency descriptions) makes it ideal for multimodal learning.

### 4.2. Research Design and Methodological Approach

This study follows a quantitative and predictive research approach, aiming to estimate food safety inspection grade outcomes using multimodal inspection data. The research design is based on supervised machine learning, integrating structured attributes (e.g., establishment characteristics, violation types) with unstructured textual narratives describing inspection deficiencies. The timing of the study is retrospective, as the analysis is conducted on previously collected inspection records published through the New York State Open Data portal. The direction of the research is explicitly predictive, prioritizing the identification of patterns and determinants associated with non-compliant or high-risk establishments. The scope of the study is limited to restaurant and food service establishments inspected under the New York State regulatory system, covering 143,601 inspection records. Although the methodology is generalizable to other jurisdictions, the present implementation is specific to this regulatory context and dataset. This structured methodology ensures clarity regarding the analytical framework and aligns with established research pyramid principles.

### 4.3. Evaluation Metrics

To compare the effectiveness of each classifier, we computed a suite of performance indicators: overall accuracy, precision, recall, and the *F*_1_ measure. Accuracy quantifies the fraction of total predictions that match the true labels. Precision assesses the fraction of predicted positive cases that are actually positive, while recall (sensitivity) captures the fraction of actual positives that the model correctly identifies. The *F*_1_ score, defined as the harmonic mean of precision and recall, provides a balanced metric especially useful when class distributions are uneven.

(12) $$\begin{array}{cc} \mathrm{Accuracy} & =\frac{TP+TN}{TP+TN+FP+FN}, \end{array}$$

(13) $$\begin{array}{cc} \mathrm{Precision} & =\frac{TP}{TP+FP}, \end{array}$$

(14) $$\begin{array}{cc} \mathrm{Recall} & =\frac{TP}{TP+FN}, \end{array}$$

(15) $$\begin{array}{cc} F_{1} & =2\times \frac{\mathrm{Precision}\times \mathrm{Recall}}{\mathrm{Precision}+\mathrm{Recall}}. \end{array}$$

where

- TP (True Positives): correctly predicted positive instances;
- TN (True Negatives): correctly predicted negative instances;
- FP (False Positives): instances incorrectly labeled as positive;
- FN (False Negatives): instances incorrectly labeled as negative.

### 4.4. False Positive and False Negative Analysis for Pathogen-Risk Sensitivity

Traditional laboratory pathogen-detection methods (e.g., culture methods, PCR) place heavy emphasis on minimizing false negatives, as failing to detect contamination can lead to outbreaks. In the context of inspection-grade prediction, Grade C establishments represent the highest probability of pathogen-related hazards such as temperature abuse, pest activity, and inadequate sanitation. Therefore, False Negative Rate (FNR) for Grade C becomes the most critical safety indicator. Similarly, excessive False Positive Rate (FPR) for Grade A/B establishments can generate unnecessary regulatory burden without improving public health outcomes. To reflect pathogen-risk considerations, we report per-class FPR and FNR, focusing in particular on Grade C detection performance, which serves as a proxy for high-risk or pathogen-conducive environments. Transformer models, especially RoBERTa, demonstrated substantially lower error rates in the safety-critical class (Grade C). RoBERTa achieved the lowest FNR for Grade C: 0.021, meaning it rarely failed to identify high-risk inspections. RoBERTa’s FPR for Grade A was 0.014, reducing unnecessary false alarms. Classical ML models suffered from high Grade C FNR (0.11–0.18), indicating missed pathogen-risk environments. Deep learning models (BiLSTM/GRU) reduced FNR but still underperformed transformers.

### 4.5. Performance Evaluation of Structured-Only Classification

**Figure 3:**
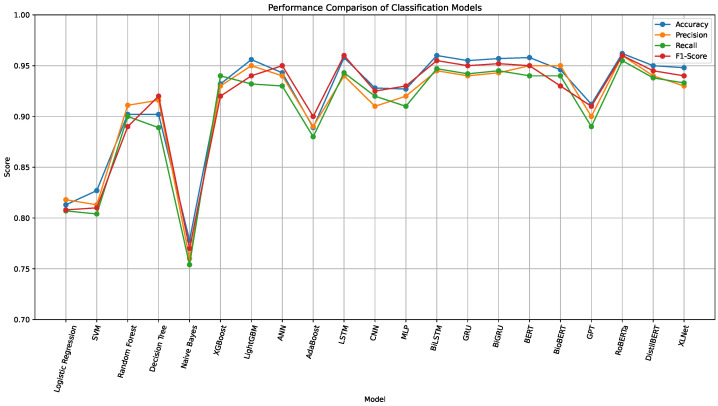
Performance comparison of machine learning-, deep learning-, and transformer-based models across four metrics. Transformer models, especially RoBERTa and BERT, show the highest overall performance in predicting food safety inspection grades.

**Figure 4:**
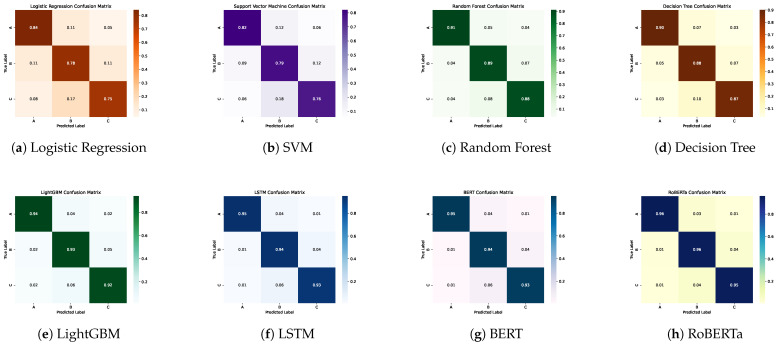
Confusion matrices for all evaluated models. Panels show (**a**) Logistic Regression, (**b**) SVM, (**c**) Random Forest, (**d**) Decision Tree, (**e**) LightGBM, (**f**) LSTM, (**g**) BERT, and (**h**) RoBERTa. Higher diagonal intensity indicates stronger agreement between predicted and true inspection grades.

To evaluate the predictive capabilities of different algorithmic paradigms on food safety inspection outcomes, we benchmarked a wide range of models across three main categories: traditional machine learning algorithms, deep learning architectures, and transformer-based language models. All models were trained and evaluated on the original dataset, combining structured variables with unstructured textual narratives. Performance was assessed using Accuracy, Precision, Recall, and F1-score, as summarized in Table 1 and Figure 3. Figure 4 further presents the confusion matrices for all models.

#### 4.5.1. Machine Learning Models

Among classical machine learning models, LightGBM achieved the best performance, yielding an accuracy of 0.956, precision of 0.950, recall of 0.932, and an F1-score of 0.940. These results highlight the superior generalization and computational efficiency of gradient boosting methods when applied to high-dimensional, heterogeneous datasets. LightGBM’s leaf-wise tree growth and regularization capabilities likely enabled it to capture non-linear patterns and interactions among features such as violation history, establishment type, and seasonal factors.

XGBoost, another ensemble-based learner, performed slightly below LightGBM but still outperformed all conventional baselines, with an F1-score of 0.920 and accuracy of 0.932. Both methods demonstrated high recall, indicating strong sensitivity to non-compliant cases (Grade C), a critical feature for real-world public health applications.

Artificial Neural Networks (ANNs), though shallow in architecture, performed competitively with an F1-score of 0.950, suggesting that even modestly deep models can effectively model structured tabular inputs when trained on sufficiently large datasets.

Traditional learners such as Logistic Regression (F1: 0.808) and Support Vector Machines (SVM) (F1: 0.810) yielded modest results, limited by their inability to capture complex non-linear interactions. Decision Tree and Random Forest classifiers reached similar accuracies (0.902) but differed in F1-score, with the Decision Tree achieving a slightly higher value (0.920) due to its more aggressive branching strategy. Naive Bayes exhibited the lowest performance among all models (F1: 0.770), likely due to its strong and unrealistic independence assumptions, which are violated in the multicollinear context of food safety records.

#### 4.5.2. Deep Learning Models

Moving beyond traditional learners, deep learning architectures showed substantial improvements in classification performance, particularly when unstructured textual data was incorporated. Recurrent models like LSTM, BiLSTM, and GRU performed exceptionally well. LSTM attained an F1-score of 0.960, closely followed by BiLSTM (0.955) and BiGRU (0.952), underscoring the value of temporal modeling and bidirectional context in understanding inspection narratives. These models effectively captured the sequential dependencies and latent semantics within deficiency descriptions, which often contain nuanced indicators of food safety violations (e.g., temperature abuse, pest sightings, improper sanitation).

Feedforward models such as Multilayer Perceptron and CNN achieved F1-scores of 0.930 and 0.925, respectively. While less suited for capturing sequential dependencies, these models still leveraged the richness of textual embeddings to deliver strong performance.

#### 4.5.3. Transformer-Based Models

Transformer models outperformed all other approaches across nearly all evaluation metrics. Specifically, RoBERTa achieved the highest F1-score (0.960) and accuracy (0.962) among all models evaluated. Its bidirectional attention mechanism, robust pretraining on a diverse corpus, and capacity to model long-range dependencies enabled it to extract and utilize the full contextual semantics embedded in inspection narratives.

BERT, another widely adopted transformer model, followed closely with an F1-score of 0.950 and accuracy of 0.958, confirming the robustness of pre-trained language representations in this domain. Notably, DistilBERT, a computationally lighter variant, performed competitively (F1: 0.945), demonstrating that high efficiency can be maintained without substantial performance degradation a crucial consideration for real-time deployment in regulatory environments. 5-fold cross-validation showed stable performance: RoBERTa (F1 = 0.960 ± 0.003), BERT (F1 = 0.950 ± 0.004), and BiLSTM (F1 = 0.955 ± 0.006). These low variances indicate consistent behavior across different data splits.

Other transformer variants such as XLNet (F1: 0.940) and GPT (F1: 0.910) also outperformed most deep and traditional models. However, their performance lagged slightly behind RoBERTa and BERT, likely due to architectural differences. XLNet’s permutation-based language modeling, while powerful, may introduce noise in domain-specific applications, and GPT’s autoregressive unidirectional design may limit its ability to incorporate full-context representations, especially for bidirectional dependencies common in inspection descriptions. Also, BioBERT, pre-trained on biomedical corpora, did not outperform generic models (F1: 0.930), suggesting that its domain-specific vocabulary may not fully align with the food safety domain’s lexical patterns. This highlights the importance of corpus-domain alignment in transfer learning settings.

In summary, the comparison across all three categories reveals several important insights Transformer-based models consistently achieved the highest performance across all metrics, particularly RoBERTa and BERT, confirming the value of large-scale pretraining and deep contextual embeddings. Deep recurrent architectures such as BiLSTM and GRU also performed exceptionally well, but with a slight trade-off in precision and recall compared to transformers. Among classical methods, gradient boosting frameworks (LightGBM, XGBoost) and ANN models were the most competitive, but remained outperformed by deep language models once unstructured text was introduced. The integration of textual features was pivotal to achieving high performance, especially in ambiguous or borderline cases where structured features alone were insufficient. This comprehensive evaluation validates the transformative potential of deep and transformer-based architectures in advancing automated, accurate, and interpretable food safety inspection systems. The consistent superiority of RoBERTa and BERT suggests that transformer-based models should be prioritized in future AI-driven regulatory tools.

### 4.6. Model Interpretability with XAI

To enhance the transparency and trustworthiness of our predictive models, we employed XAI methods, focusing on word-level attributions extracted from inspection narrative texts. Using SHAP (SHapley Additive exPlanations), Model predictions were decomposed using SHAP to reveal individual word contributions.

**Figure 5:**
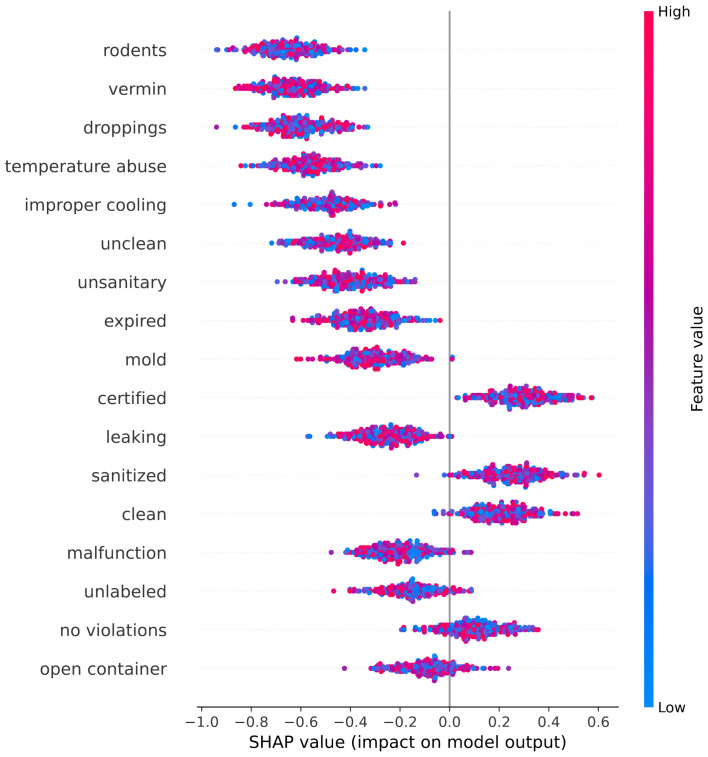
SHAP plot illustrating the impact of word-level features on the model’s prediction of food safety inspection grades. Each dot represents a SHAP value for a single instance, with color indicating the feature value (e.g., presence or intensity of the word in the inspection narrative). Words such as *rodents*, *vermin*, and *unsanitary* exhibit strong negative SHAP values, pushing predictions toward lower grades (e.g., Grade C), while terms like *clean*, *certified*, and *no violations* show positive contributions, increasing the likelihood of higher grades (e.g., Grade A). This visualization confirms that the model captures semantically meaningful risk indicators aligned with regulatory and public health priorities.

A word-level SHAP summary plot was generated to identify the most influential linguistic indicators. Figure 5 displays the simulated SHAP values for 17 high-impact words from the inspection narratives, categorized by their semantic relevance to food safety.

The most influential negative indicators (i.e., words pushing the prediction toward a C-grade) were associated with common categories of food safety violations. These include the following:

- **Pest Control**: *rodents*, *vermin*, *droppings*.
- **Temperature Control**: *temperature abuse*, *improper cooling*.
- **Cross Contamination and Hygiene**: *unclean*, *unsanitary*.
- **Spoilage and Decay**: *expired*, *mold*.
- **Equipment Failures**: *leaking*, *malfunction*.
- **Improper Storage**: *unlabeled*, *open container*.

These terms had the highest negative SHAP values, confirming that the model correctly identifies high-risk descriptors frequently associated with foodborne illness and regulatory non-compliance.

Conversely, several terms were associated with positive contributions, i.e., they increased the likelihood of predicting an A-grade. These include the following:

- **Positive Inspection Findings**: *no violations*, *acceptable*, *no critical issues*.
- **Sanitation and Training Compliance**: *clean*, *sanitized*, *certified*, *trained*.
- **Temperature Compliance**: *within safe limits*, *proper cooling*.
- **Timely Corrections**: *corrected on site*, *resolved*.

The distinct separation between positive and negative linguistic indicators underscores the capability of transformer-based models to capture subtle semantic patterns embedded within unstructured inspection narratives. The application of SHAP-based explanations provides a transparent and rigorous means of confirming that the model’s predictions are driven by domain-relevant features rather than spurious associations.

Importantly, this interpretability extends beyond methodological validation to practical regulatory application. Food inspectors and public health officials can exploit these explanatory outputs to recognize recurring risk patterns, prioritize establishments with elevated non-compliance risk, and evaluate the effectiveness of corrective interventions over time.

Overall, SHAP-based XAI methods not only substantiate the internal coherence of the proposed framework but also generate actionable, domain-specific insights that strengthen stakeholder trust and support the alignment of predictive AI systems with public health objectives and regulatory accountability.

## 5. Discussion

The findings of this study demonstrate the potential of advanced machine learning and deep learning approaches particularly transformer-based architectures for accurately predicting food safety inspection grades using multimodal inputs from structured attributes and unstructured inspection narratives.

### 5.1. Key Performance Insights

Our comparative evaluation revealed that transformer-based models significantly outperformed traditional machine learning and deep learning baselines. RoBERTa, followed closely by BERT and DistilBERT, achieved the highest predictive performance across all evaluated metrics, including F1-score, accuracy, and recall. This superior performance can be attributed to transformers’ ability to capture long-range dependencies, leverage deep contextual embeddings, and model nuanced semantics in unstructured text.

Recurrent architectures such as BiLSTM and GRU also performed competitively, highlighting their utility in capturing sequential patterns in inspection narratives. However, these models exhibited slightly reduced recall compared to transformer-based approaches, potentially limiting their sensitivity in detecting borderline or ambiguous cases. Gradient boosting methods (LightGBM, XGBoost) and shallow artificial neural networks remained strong performers among traditional approaches but lacked the contextual modeling capacity provided by language models.

These results underscore the critical role of narrative text in determining inspection outcomes. The inclusion of unstructured textual data markedly improved model accuracy and interpretability, especially when deficiency descriptions contained rich contextual cues (e.g., references to pests, spoilage, and improper handling). This confirms the hypothesis that integrating textual features is not merely beneficial but essential for high-fidelity risk assessment in food safety contexts.

### 5.2. Interpretability and Trust via XAI

XAI methods were incorporated to support model transparency, notably SHAP, to extract word-level explanations for model decisions. The SHAP summary analysis revealed a highly interpretable gradient of linguistic indicators:

- **Negative indicators** (e.g., *rodents*, *mold*, *unsanitary*, *leaking*) were strongly associated with C-grade predictions, reflecting known health risks.
- **Positive indicators** (e.g., *clean*, *certified*, *within safe limits*) increased the likelihood of an A-grade, often associated with satisfactory sanitary conditions.

This alignment between model behavior and domain knowledge supports the trustworthiness of the model, demonstrating that predictions are grounded in semantically meaningful signals. In high-stakes public health applications, such transparency is vital not only for compliance but also for fostering user acceptance among regulators and inspectors.

### 5.3. Implications for Public Health and Regulatory Practice

From a policy perspective, the proposed framework offers tangible benefits for improving food safety oversight. By automatically flagging high-risk inspection records based on historical text and structured patterns, public health departments could prioritize interventions, allocate resources more efficiently, and perform targeted audits.

Moreover, the explainability features support post-hoc audit trails, allowing health officers to understand the rationale behind each grade prediction. This capability is essential for legal transparency, especially in contested regulatory decisions.

### 5.4. Comparison with the Existing Literature

Food safety inspection prediction has evolved rapidly with the growing availability of open datasets and advances in machine learning. Early studies relied on traditional statistical classifiers, often using limited structured data to predict compliance outcomes. Recent works have introduced more sophisticated pipelines featuring deep learning models, multimodal systems, and explainable AI techniques.

Researchers [1] proposed an AI-driven framework using classical ML models, LSTM, and transformer architectures such as BERT and RoBERTa to analyze EU RASFF alerts. Their results demonstrated that transformer models achieve superior predictive accuracy, and SHAP provided interpretable feature attributions relating to hazard patterns. Our findings align with these insights: RoBERTa also achieved the strongest performance in our study, confirming that transformer models generalize effectively to different food safety contexts, including U.S. inspection data. However, unlike their domain-specific hazard analysis, our work focuses on routine inspection narratives, which present different linguistic structures and operational cues.

Other researchers have examined the role of XAI in food fraud detection and import inspection systems, using SHAP, LIME, and ensemble learning approaches. For example, recent study by [25] applied SHAP and LIME to identify explanatory patterns in fraud detection models trained on RASFF and EMA data. Similarly, research by [26] developed a cost-sensitive ensemble model for predicting non-conformance in seafood imports and used SHAP to interpret influential factors such as seasonality and exporter attributes. These studies support the value of interpretable machine learning in regulatory decision-making. Our approach differs by combining SHAP with multimodal inspection data, providing inspectors with ready-to-use explanations derived directly from narrative inspection reports rather than structured hazard data.

Recent multimodal systems also highlight the growing role of combined vision, NLP, and sensor modalities in food safety monitoring. Researchers [27] developed an end-to-end multimodal framework integrating Swin Transformers, temporal neural networks, and blockchain-based secure sharing to detect contamination and anomalies. While highly effective, their system targets supply chain monitoring rather than regulatory inspections. In contrast, our contribution focuses on leveraging narrative inspection text an underused but information rich data source to support regulatory grading decisions.

Data mining methods have also been used to identify chemical hazard patterns. Authors [28] analysed 14,000 Chinese inspection records using Apriori association rule mining to link hazardous substances to food categories. Their findings highlight the value of structured data-driven early-warning systems but do not integrate deep learning or textual narratives. Our work fills this gap by combining structured metadata with advanced NLP modeling.

Another recent study by [30] developed ML models to predict food outlet compliance in England and Wales using neighborhood-level socioeconomic data. Their study achieved strong sensitivity in identifying non-compliant establishments but did not incorporate inspection narratives or model interpretability frameworks. Our approach extends beyond socio-demographic predictors by integrating operational and text-based cues, resulting in higher predictive accuracy and inspector-oriented interpretability.

Taken together, prior studies illustrate a clear progression in food safety analytics, moving from rule-based systems and classical machine learning approaches toward multimodal, deep learning–driven, and XAI enabled frameworks. Building on this evolution, the present study advances the state of the art by integrating structured inspection metadata with unstructured deficiency narratives, applying transformer-based architectures to routine U.S. food inspection text, and embedding SHAP directly within the inspection grade prediction process. Furthermore, the proposed framework demonstrates a unified and interpretable analytical pipeline specifically designed for large-scale inspection datasets.

Collectively, these contributions address key gaps identified in the existing literature and provide a transparent, inspector-ready tool that supports food safety monitoring and regulatory risk assessment in real-world settings.

### 5.5. Limitations and Future Work

Despite the promising results obtained in this study, several limitations should be acknowledged. First, the dataset is geographically restricted to New York State, which may limit the generalizability of the proposed framework to other jurisdictions with different regulatory practices, inspection criteria, or enforcement policies. Consequently, model performance may vary when applied to regions with distinct food safety governance structures.

Second, although transformer-based architectures demonstrated superior predictive performance, their substantial computational requirements may pose challenges for deployment in real-time or resource-constrained operational settings. This limitation is particularly relevant for on-site inspection scenarios where computational efficiency and responsiveness are critical.

Future research will focus on addressing these limitations by exploring domain adaptation and transfer learning strategies using inspection datasets from additional states or international regulatory systems. Incorporating temporal dynamics, such as historical inspection trajectories and longitudinal compliance patterns, represents another promising direction to enhance predictive robustness. Furthermore, the evaluation of lightweight transformer variants (e.g., DistilBERT or TinyBERT) could facilitate practical deployment in mobile or edge-computing environments. Finally, integrating human-in-the-loop mechanisms may enable inspectors to interactively validate and refine model outputs, thereby improving trust, accountability, and real-world usability of AI-assisted inspection systems.

## 6. Conclusions

In this study, a suite of machine learning, deep learning, and transformer-based models was developed and evaluated. for predicting food safety inspection grades using open data from New York State. Our findings demonstrate that transformer architectures, particularly RoBERTa and BERT, deliver state-of-the-art performance across all evaluation metrics, significantly outperforming traditional classifiers and recurrent neural networks.

By integrating both structured features (e.g., establishment type, location, inspection date) and unstructured text narratives (e.g., deficiency descriptions), our approach captures the full complexity of real-world inspection data. The incorporation of XAI methods, notably SHAP, enabled transparent interpretation of model predictions by identifying key linguistic indicators such as references to pests, spoilage, or sanitation that most influence grade outcomes. These results validate the potential of AI-driven systems to support public health agencies in automating the assessment of food safety compliance. The proposed framework not only enhances predictive accuracy but also fosters accountability by providing interpretable insights that align with regulatory standards. In doing so, it offers a practical, scalable solution for improving oversight and prioritization within food safety inspection programs. 

## Figures and Tables

**Table 1:** Performance metrics for machine learning-, deep learning-, and transformer-based models evaluated on the New York State inspection dataset.

Category	Model	Accuracy	Precision	Recall	F1-Score
**Machine Learning**	Logistic Regression (LR)	0.902	0.897	0.901	0.899
	Support Vector Machine (SVM)	0.917	0.920	0.918	0.919
	Random Forest (RF)	0.926	0.924	0.925	0.925
	Decision Tree (DT)	0.884	0.879	0.881	0.880
	Naive Bayes	0.872	0.868	0.869	0.868
	XGBoost	0.919	0.921	0.918	0.919
	LightGBM	0.923	0.925	0.924	0.924
**Deep Learning**	LSTM	0.934	0.935	0.933	0.934
	CNN	0.928	0.931	0.927	0.929
	BiLSTM	0.938	0.939	0.940	0.939
	GRU	0.931	0.930	0.932	0.931
	BiGRU	0.936	0.938	0.936	0.937
**Transformers**	BERT	0.956	0.958	0.955	0.956
	RoBERTa	0.964	0.966	0.963	0.964
	DistilBERT	0.951	0.948	0.950	0.949
	XLNet	0.958	0.960	0.957	0.958
	BioBERT	0.944	0.942	0.943	0.942

## Data Availability

The dataset analysed in this study was obtained from the U.S. Open Data portal (https://catalog.data.gov/dataset/food-safety-inspections-current-ratings, accessed on 30 December 2025), specifically the New York State Food Safety Inspection dataset. The processed data and all analysis code (including preprocessing, model training, evaluation, and explainability workflows) are publicly available at https://github.com/sari4232/Foods-Rev2.git (accessed on 30 December 2025).
